# Root colonization by the endophytic fungus *Piriformospora indica* shortens the juvenile phase of *Piper nigrum* L. by fine tuning the floral promotion pathways

**DOI:** 10.3389/fpls.2022.954693

**Published:** 2022-11-09

**Authors:** R. S. Lekshmi, S. Sora, K. N. Anith, E. V. Soniya

**Affiliations:** ^1^ Division of Transdisciplinary Biology, Rajiv Gandhi Centre for Biotechnology, Thiruvananthapuram, Kerala, India; ^2^ Department of Agricultural Microbiology, College of Agriculture, Kerala Agricultural University, Thiruvananthapuram, Kerala, India

**Keywords:** piriformospora indica, piper nigrum, flowering induction, phytohormones, juvenile phase

## Abstract

*Piriformospora indica*, the mutualistic biotrophic root colonizing endosymbiotic fungus belonging to the order Sebacinales, offers host plants various benefits and enhances its growth and performance. The effect of colonization of *P. indica* in *Piper nigrum* L. cv. Panniyur1 on growth advantages, floral induction and evocation was investigated. Growth and yield benefits are credited to the alteration in the phytohormone levels fine-tuned by plants in response to the fungal colonization and perpetuation. The remarkable upregulation in the phytohormone levels, as estimated by LC- MS/MS and quantified by qRT-PCR, revealed the effectual contribution by the endophyte. qRT-PCR results revealed a significant shift in the expression of putative flowering regulatory genes in the photoperiod induction pathway (*FLOWERING LOCUS T, LEAFY, APETALA1, AGAMOUS, SUPPRESSOR OF CONSTANS 1, GIGANTEA, PHYTOCHROMEA*, and *CRYPTOCHROME1*) gibberellin biosynthetic pathway genes (*GIBBERELLIN 20-OXIDASE2, GIBBERELLIN 2-OXIDASE, DELLA* PROTEIN *REPRESSOR OF GA1-3 1*) autonomous (*FLOWERING LOCUS C, FLOWERING LOCUS VE, FLOWERING LOCUS CA*), and age pathway (*SQUAMOSA PROMOTER LIKE9, APETALA2*). The endophytic colonization had no effect on vernalization (*FLOWERING LOCUS C*) or biotic stress pathways (*SALICYLIC ACID INDUCTION DEFICIENT 2, WRKY family transcription factor 22*). The data suggest that *P. nigrum* responds positively to *P. indica* colonization, affecting preponement in floral induction as well as evocation, and thereby shortening the juvenile phase of the crop.

## Introduction

The “king of spices,” black pepper, is the most widely grown spice in the world. Its biological role has been thoroughly examined by Ahmad et al. (2012). It contains alkaloids, mainly piperamides, which have a wide range of therapeutic applications. It has antimutagenic and antioxidant properties in addition to tumour inhibitory properties. The alkaloid piperine confers the pungency and, the essential oil accords the aroma of black pepper (Srinivasan, 2007). *P. nigrum* is a perennial plant with a prolonged vegetative phase of over 3-4 years to reach the flowering stage and that results in a lengthy waiting period for the farmers. Plant growth experiments in black pepper are challenging due to the perennial character of the crop and the practical difficulty of growing it under artificial conditions. Under greenhouse circumstances, however, miniaturised black pepper plants known as “bush pepper” could be utilised to conduct research. They are propagated from the non-conventional planting material, i.e., the lateral shoots (plagiotrophs) of mother vines. Since they are clonally propagated, they inherit the true-to-type characters of the mother vine. Unlike the main crop, bush pepper plants do not trail up, instead they spread laterally and could be maintained in pots in a greenhouse.

**Figure 1:**
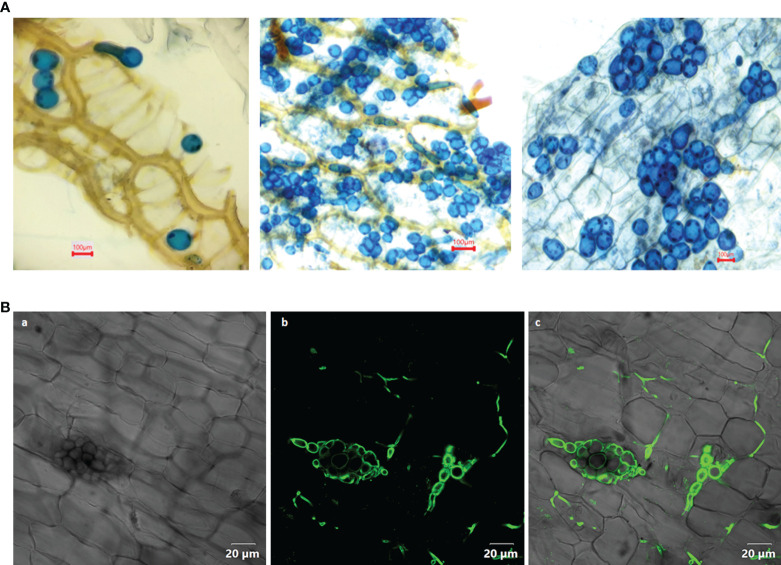
**(A)** Colonization of *Piriformospora indica* in the root cortical cells of *Piper nigrum* L. cv. Panniyur 1. Pear shaped chlamydospores were visualized at 20^th^ DAI under 40x, bright field microscope (euromex model no: IS.1153-EPL-DF, Holland). **(B)** Confocal analysis (OLYMPUS FV3000, Tokyo, Japan) of *P. indica* spores stained with WGA-AF 488, indicating its colonization in the roots of *P. nigrum* 20 days post co-cultivation. (a) Bright field image (b) WGA-AF 488 stained spores and intercellular hyphae excited at 488 nm (c) Overlay with bright field image.

**Figure 2:**
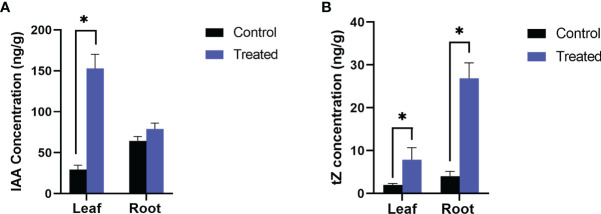
Endogenous level of growth hormones, **(A)** IAA and **(B)** Trans zeatin (tZ) in *P. indica* colonized and non-colonized plants. Samples are taken 60^th^ DAI. Hormone quantification was carried out on the basis of peak area ratio with the hormone standards using LC/MS. Each value is the mean ± SE of three biological replicates. Asterisks indicate significance at * P < 0.05 by student *t*- test.

*Piriformospora indica*, belonging to Basidiomycotina (family: sebacinaceae), was discovered by Verma et al. (1998). It is a root endophytic fungus with an expansive host range and favourably cultured axenically in a standard culture medium (**Figure 1A** and **Supplementray Figure 2**). A perusal of literature has revealed the fact that the endophyte has tremendous potential for promoting plant growth. It has been reported to boost the biomass of the plant, induce early flowering response and augment the yield of crop plants (Verma et al., 1998; Vadassery et al., 2009; Achatz et al., 2010; Das et al., 2012; Anith et al., 2018). Apart from that, it promotes nutrient uptake and transport, enables plants to survive temperature, salt and drought stresses, accord systemic resistance to biotic stress, as well as to toxins and heavy metals (Sherameti et al., 2005; Waller et al., 2005; Sherameti et al., 2008; Fakhro et al., 2009; Kumar et al., 2009; Yadav et al., 2010; Athira and Anith, 2020). *P. indica* has an extensive host range, indicating that the mutualist has developed effective and efficient colonization strategies. The role of phytohormones and recruitment of signalling pathways by the endophytes in the successful establishment of symbiosis has been well documented (Vadassery et al., 2008; Schafer et al., 2009; Lee et al., 2011; Hilbert et al., 2012). Beneficial endophytic microorganisms manipulate the plant hormone levels to get in compromise with the innate immune responses for its easy establishment. The production of phytohormones such as auxin, cytokinin, and gibberellic acid is thought to be responsible for the increased growth and yield (Varma et al., 2012; Gill et al., 2016). Experiments were conducted to study the changes in the level of growth and defence phytohormones in the colonized and non-colonized plants at molecular level.

Flowering response in plants is co-ordinated by both internal (flowering regulatory pathways) and external (environmental) cues that switch them from vegetative to reproductive stage, which is inevitable for crop production. Studies have proved that five distinct developmental pathways guide floral transition in *Arabidopsis* (Pan et al., 2017). Among them are the genes that control the photoperiod, the vernalization, gibberellin biosynthesis, signalling, the autonomous and the age pathways (Pan et al., 2017). In general, the regulatory pathways that control the flowering time are conserved across the plant kingdom. Floral evocation is influenced by a set of genes that can be classified as meristem identity genes and floral organ identity genes (Blazquez and Weigel, 2000). *P. indica* has been reported to promote growth, leading to preponement in floral response in *Coleus forskohill* (Das et al., 2012) and in *Arabidopsis thaliana* (Kim et al., 2017). Colonization of black pepper roots by *P. indica* was reported previously and was found to boost plant development and increase secondary metabolite content in the berries (Anith et al., 2015; Anith et al., 2018). The goal of this study was to figure out the molecular mechanism behind the endophytic relationship between *P. indica* and *P. nigrum* with special emphasis on flowering response. In fact, this is the first attempt to study the impact of *P. indica* on the perennial spice crop black pepper at molecular level. To shed light on the influence of root colonization of the fungus in the floral transition response of black pepper, they were co-cultivated with the endophytic fungus and the gene regulation pathways were analysed.

## Materials and methods

### Preparation of the planting material for root infection with P. indica inoculum

The bush pepper plantlets were made from the lateral vines of a healthy mother plant of the black pepper variety Panniyur I. Rooted cuttings for the experiment were obtained from The Black pepper farm, Kerala Agricultural University, Vellanikkara, Thrissur, Kerala, India. The rooted cuttings (three to four leaf stage) were carefully transferred to pots (30 cm diameter, 40 cm height) filled with potting media (soil: sand: farm yard manure, 1:1:1) (pH6.5). The plantlets were root inoculated at the time of transplantation.

### Mass multiplication and inoculation of plants with the fungal endophyte

The fungus *P. indica* obtained from the Department of Agricultural Microbiology, College of Agriculture, Kerala Agricultural University, Vellayani, Kerala, India, was cultured in Potato Dextrose broth (PDB pH 6.5) in Erlenmeyer flasks (500mL) at 25°C in an incubator shaker (120 r min^-1^) for 14 days. Then the mycelia were filtered using a muslin cloth and washed thoroughly using sterile water. The inoculum was produced at a final concentration of one percent (w/v) in sterile vermiculite as per the method suggested by Anith et al. (2011).

The experimental plants were appropriately irrigated and maintained in the greenhouse under natural light with 25 per cent shade. Two per cent water-soluble chemical fertilizer (N:P:K:: 17:17:17) was provided once in 30 days at a rate of 100 ml per plant. Rooted cuttings were inoculated by adding 50 g of the prepared inoculum to the potting medium. The cuttings (15 replicates) were transplanted to the pots in such a way that the inoculum made direct contact with the roots. The experiment was performed in a completely randomised design, with the uninoculated control (15 replicates) plants been maintained, by the addition of an equal quantity of sterile vermiculite while planting (Anith et al., 2018). Expression of flowering-regulatory genes was measured 30, 40, 50, and 60 days after inoculation (DAI).

### Examination of root colonization

Root colonization was examined from the 10^th^ DAI onwards. The roots of treated and untreated plants were sampled without damaging them. After washing the roots with double distilled water, the roots were cut into one cm long fragments and softened by boiling in KOH (10%) solution for 5 minutes. The root bits were then acidified for 5 minutes with 1M HCl and stained for 15 minutes in lactophenol-trypan blue (0.02%). De-staining was done using lactophenol solution and the fungal colonization was assessed by noting the presence of chlamydospores in the roots at 40x, bright field microscope (euromex model no: IS.1153-EPL-DF, Holland).

Wheat germ agglutinin- Alexa Flour 488 (WGA-AF 488) staining was performed with slight changes in the procedure mentioned by Satheesan et al. (2012). The co-cultivated roots were fixed using 0.15 per cent trichloroacetic acid and washed in 1X phosphate buffer saline (pH 7.4). The roots were neutralized in 1X PBS after boiling with 10 per cent KOH for one minute. The root tissue were then stained for 6 hours using WGA-AF 488 (100µg ml^-1^) (Invitrogen, Oregon, USA) facilitated by vacuum-infiltration for one minute thrice at 50 mmHg. The root tissue were destained overnight in 1X PBS. The conjugated stain was excited at 488 nm and detected using Confocal microscope (OLYMPUS FV3000, Tokyo, Japan) at 500-600 nm.

The presence of *P. indica* in the roots was further confirmed by molecular detection. Roots of treated and untreated plants were collected 30 days post inoculation. They were thoroughly cleansed with double distilled water followed by (0.1%) DEPC- treated autoclaved water. Plant RNA isolation kit (Spectrum, SIGMA) was used to isolate total RNA. cDNA was prepared using Bio-Rad, iScript™ Select cDNA synthesis kit. The colonization was verified by performing qRT-PCR reactions (Thermo Fisher Scientific Power SYBR™ Green PCR Master Mix) using primers specific for *P. indica* - *transcription elongation factor* (Pi*-TEF*) gene.

### Estimation of growth and defense phytohormones

The growth phytohormones, zeatin (IPT) and indole-3-acetic acid (IAA) from root and leaf samples of treated and untreated plants, were extracted according to Šimura et al. (2018) with modifications. LC-MS/MS was performed according to a published protocol (Schäfer et al., 2013) with minor modifications. The defense phytohormones, salicylic acid (SA), jasmonic acid (JA), JA-Ile, cis-OPDA and abscisic acid (ABA) were estimated using root sample according to the modified procedure reported by Vadassery et al., 2012. Quantification of the hormones relative to the signals of their corresponding internal standards were performed.

### RNA isolation and analysis of relative gene expression by qRT-PCR

Total RNA was extracted from the tender apical leaves of both inoculated and control plants (one leaf per plant/biological replicate) collected at 30, 40, 50 and 60 DAI, using a Plant RNA isolation kit (Spectrum, SIGMA, Germany). For cDNA preparation 1μg of RNA was taken and reverse transcribed using Bio-Rad (USA), iScript™ Select cDNA synthesis kit according to the manufacturer’s protocol. qRT-PCR of representative genes in flowering regulatory pathways were performed using 7900HT Fast Real Time PCR (Applied Biosystems, USA). For amplification, the Thermo Fisher Scientific Power SYBR™ Green PCR Master Mix was used (optimum annealing temperature was 58°C for all the primers). To calculate the relative gene expression, the 2^-ΔΔCt^ method was used (Livak and Schmittgen, 2001). The gene-specific primers used for qRT-PCR analysis are listed in **Supplementary Table 1**. Primers specific for the constitutively expressed, house-keeping gene actin was used as endogenous control.

### Statistical analysis

Quantitative real-time PCR was performed and the 2^-ΔΔCt^ (Livak and Schmittgen, 2001) was used to calculate the cycle threshold value of each sample. The primers used for the qRT-PCR experiment were designed using Primer3 (v.0.4.0) https://bioinfo.ut.ee/primer3-0.4.0/ software. The expression level of the representative genes was normalized to *actin2.* Three biological replicates were analysed with three technical replicates for each. Student’s two-tailed *t*- test was used to determine significance of the gene expression data in this study.

## Results

### Root colonization

In the root cortical cells, pear-shaped chlamydospores were observed on 20^th^ DAI (**Figures 1A, B**). This was considered as a preliminary indication of root colonization. No spores could be visualized in control plants. The qRT-PCR analysis of the inoculated plants showed a significant expression (2.5 - fold) of the Pi-*tef* (transcription elongation factor) gene.

### Flowering time

In this study it was observed that the endophytic association of *P. indica* pushed forward the floral response reaction in black pepper. Four to five spikes emerged in the inoculated plants after 60 days of transplantation, whereas no spike emerged in the control plants. Inoculated plants had eight to ten spikes after four months of transplantation, but control plants had none. Two to three spikes formed twelve months after transplantation in control plants, and about 20-30 percent of the spikes dropped down without berry formation, as well as premature dropping of berries was also observed. Plant growth was found accelerated when germinated seedlings were transplanted to inoculated potting mixture (**Supplementary Figure 1**).

### Estimation of growth and defense phytohormones

**Figure 3:**
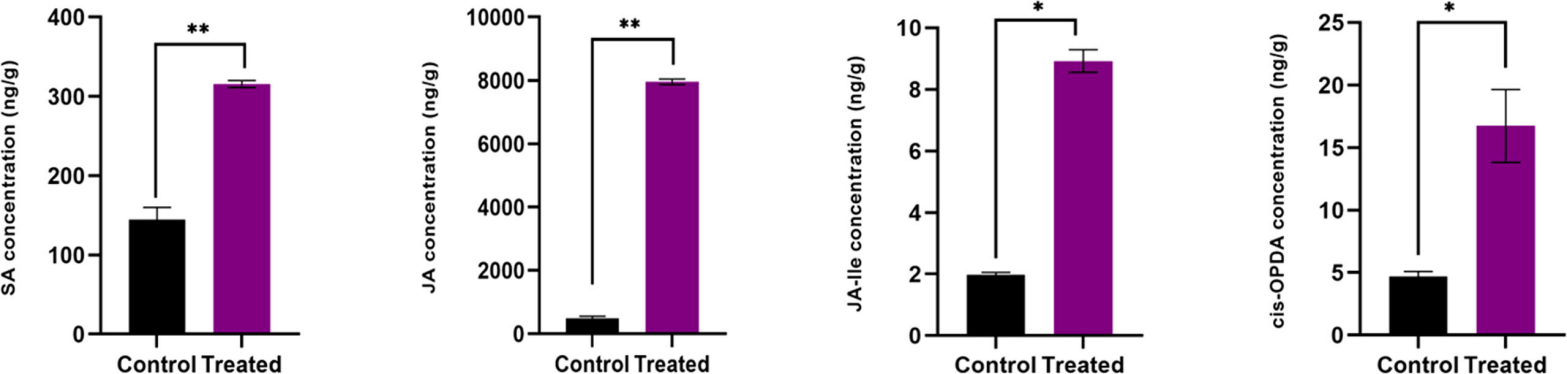
Endogenous level of defense phytohormones SA, JA, JA-Ile and cis-OPDA, in *P. indica* colonized and non-colonized plants. Samples are taken 60th DAI. Hormone quantification was carried out on the basis of peak area ratio with the hormone standards using LC/MS. Each value is the mean ± SE of three biological replicates. According to student *t*- test, asterisks indicate significance at ** P < 0.01 and * P < 0.05.

**Figure 4:**
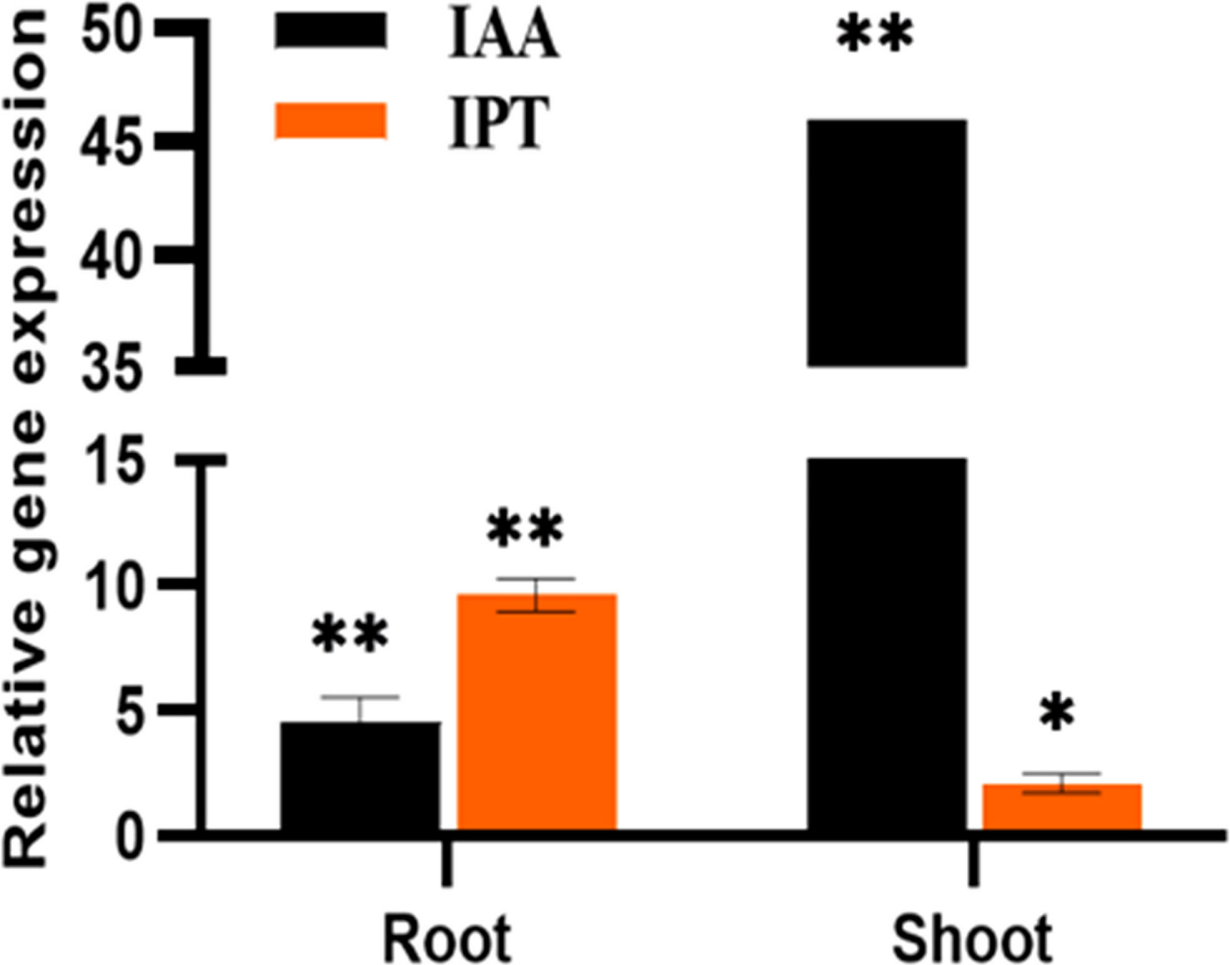
The relative expression of phytohormones, IAA and IPT genes of *P. indica* colonized and non-colonized plants 60 DAI. Data based on mean fold changes ± SE of three biological replicates. Asterisks indicate significance at ** P < 0.01 and * P < 0.05 by student *t*- test.

In the inoculated plants the concentration of growth (**Figures 2A, B**) and defense phytohormones (**Figure 3**) were significantly high as estimated using LC-MS/MS and an upregulation in the expression of genes corresponding to IAA and IPT was also observed in the inoculated plants (**Figure 4**).

### Relative expression of the flowering-regulatory genes

**Figure 5:**
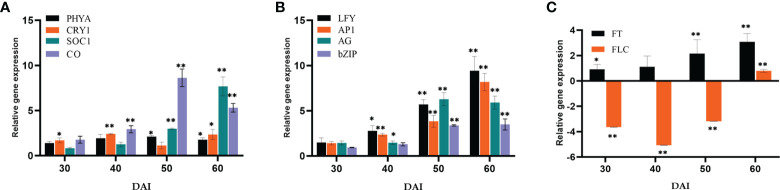
Relative gene expression of representative genes in the photoperiod pathway **(A)** *PHYA*, *CRY1*, *SOC1* & *CO*, **(B)** *LFY*, *AP1*, *AG* & *bZIP*, **(C)** *FT* & *FLC*, responsible for flowering response. The samples were isolated for qRT-PCR at 30, 40, 50 and 60 DAI. Error bars shows ±SEs of three biological replicates. As per student *t*- test, ** P< 0.01 and * P < 0.05.

Relative quantification of certain key representative (putative) genes in various flowering pathways has been carried out to determine the suitable course by which *P. indica* induces early flowering in *P. nigrum* on various days after root infection (DAI). The expression of flowering regulatory genes was checked at multiple time points after root inoculation. The transcript level of *Phytochrome A* (*PHYA*) in *P. indica* inoculated plants was 2.12- fold higher than that in control plants on the 50^th^ DAI. mRNA level of *Cryptochrome1* (*CRY1*), the blue light-absorbing cryptochromes which promote flowering was 2.35-fold elevated than the uninoculated control plants on the 60^th^ DAI and a simultaneous upregulation in the expression of *Constans* (*CO*) (8.63-fold) was observed on the 50^th^ DAI. A gradual elevation in the expression of the *Flowering locus T* (*FT*) gene (1.89 to 8.37-fold) and a simultaneous downregulation in the expression of the *Flowering locus C* (*FLC*) gene was noted between treated and control plants. *FD*, a *bZip* transcription factor was activated (3.48 - fold), followed by an apparent change in the expression of *Leafy* (*LFY*) (5.7 - fold) was observed on the 50^th^ DAI. *Apetala 1* (AP1) was upregulated (3.84- fold) on the 50^th^ DAI, and expression of *Agamous* (*AG*) was 6.27-fold higher on 50^th^ DAI. *Gigantea* (*GI*), that promotes photoperiodic flowering by inducing the FT gene was appreciably high (5.50-fold) from the 30^th^ DAI (**Figures 5A–C**).

**Figure 6:**
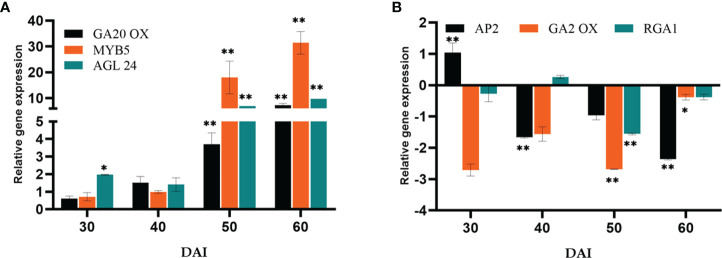
Relative gene expression of representative genes in the gibberellic acid biosynthesis pathway **(A)** *GA20OX*, *MYB5* & *AGL24* **(B)** *AP2*, *GA2OX* & RGA1, responsible for flowering response. The samples were isolated for qRT-PCR at 30, 40, 50 and 60 DAI. Error bars shows ±SEs of three biological replicates. Significance of the values were determined using student *t*- test, ** P< 0.01 and * P < 0.05.

The expression of GA biosynthesis gene *Gibberellin 20-Oxidase2* (*GA20ox2*) (positive regulator) and *GA2ox* (negative regulator) were quantified (**Figures 6A, B**). On the 50^th^ DAI, the positive regulator *GA20ox* was upregulated to 6.16-fold, and the negative regulator *GA2ox* was down-regulated. *Repressor of GA1-3 1 (RGA1)* belonging to the DELLA gene family was found to be downregulated simultaneously. *P. indica* showed its stimulatory effect on the GA responsive gene *Agamouslike 24* (*AGL24*) (1.96 to 9.74- fold) in 30 to 60 DAI and upregulation of *Supressor of overexpression of constans* (*SOC1)* (2.98 to 7.68 fold) in 40 to 50 DAI.

**Figure 7:**
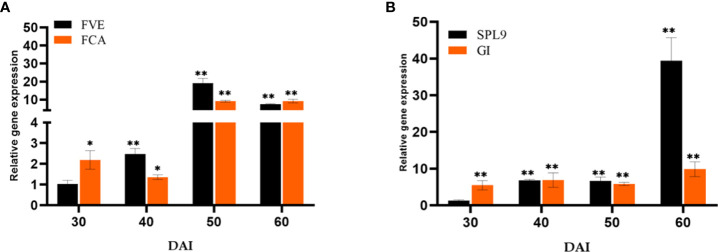
Relative expression of genes in the **(A)** autonomous pathway (FVE & FCA) and **(B)** age pathway (SPL9 & GI) respectively, responsible for floral induction. qRT-PCR was carried out with samples isolated at 30, 40, 50 and 60 DAI. Error bars shows ±SEs of three biological replicates. student *t*- test determined significance of gene expression ** P< 0.01 and * P < 0.05.

The essential Flowering time control genes, *Flowering locus VE (FVE)* and *Flowering locus CA (FCA)* involved in the autonomous pathway were quantified (**Figure 7A**). The genes *FVE* (19.05-fold) and *FCA* (9.10- fold) showed a significant response to *P. indica* on 50^th^ DAI with a simultaneous reduction in the transcript level of *Flowering locus C (FLC)*. The transcript level of *Squamosa promoter like 9 (SPL9)* was quantified and found to have a significant fold change in its expression (39.41-fold) on 60^th^ DAI with a simultaneous down regulation in the expression of *Apetala2 (AP2)* transcript, a floral repressor (**Figure 7B**). The expression level of *Agamous (AG)* was checked and was found upregulated (6.27-fold) at 50^th^ DAI. To study whether flowering is induced through a stress response pathway, the expression levels of stress responsive genes, *Salicylic acid induced deficient 2* (*SID2*), and *WRKY family transcription factor 22* (*WRKY22*) were also quantified. The mRNA level of *SID2* and *WRKY22* were comparable.

## Discussion

In the current studies axenically cultured *P. indica* was inoculated to black pepper roots to examine its effects on growth and the expression of genes controlling flowering time. *P. indica* has been reported to interfere with the biosynthesis of phytohormones and signalling to trigger growth & differentiation (Liu et al., 2019; Kundu and Vadassery, 2022), floral induction (Pan et al., 2017) and immune responses (Li et al., 2022). During colonization by the endophytes, plants try to regulate the hormone levels in the roots inorder to control their colonization and propagation. Hormones such as IAA, Zeatin, SA and jamonates have crucial roles in growth, yields, resistance to biotic and abiotic stresses and defense hormones in particular is essential for stabilizing the symbiotic association.

### Effect of *P. indica* on the phytohormone level of *P. nigrum*

The inoculated and uninoculated plants were compared by quantifying the phytohormones by LC-MS/MS. The levels of IAA, Zeatin, SA, JA, JA-Ile, cis-OPDA were significantly enhanced in *P. indica* colonized black pepper plants. Similar kind of experiments on barley (Waller et al., 2005) and maize (Kumar et al., 2009) supports our findings. Indole-3-acetic acid (IAA) which encourages cell elongation and differentiation (Asgher et al., 2015 and Ismail et al., 2021) was found to be more in plants colonized by *P. indica* which in turn must have stimulated the development of roots, evident from the enhanced formation of secondary roots and root hairs. This in turn improved the rate of shoot growth compared to the uninoculated control plants. Cytokinin on the other hand controls cell division, enhances phloem transport, promotes axillary bud growth and flowering (Kieber, 2002; Albermann et al., 2013). The enhanced levels of cytokinin in the root and shoot tissues due to the endophytic association in the current study might have contributed to improved plant growth and floral development. D'Aloia et al. (2011) also demonstrated that Arabidopsis responded to cytokinin treatment by activating the key gene (SOC1) in the floral induction pathway.

Salicylic acid (SA) plays a vital role in increasing the plants’ response to biotic (Horvath et al., 2007; Baghizadeh and Hajmo-hammadrezaei, 2011) and abiotic (Simaei et al., 2012) pressure. SA is also involved in floral induction, nutrient transfer, controls movement of stomata, protein synthesis, etc (Shah et al., 2021). Leaf health has been hypothesized as a foremost factor in photoperiodic flowering. Leaves are the important exposed plant part that can sense a diversity of environmental stimuli to initiate flowering. In the current study, upregulation in the SA content in the plant might have improved the leaf morphometrics and leaf health, thereby improving flowering induction. Healthier leaves can capture more photons, creating the primary gene signals (*via GI* and *CO*) and transmission of floral integrator gene (*FT*) to shoot apical meristem (SAM) through phloem to trigger floral evocation (Corbesier et al., 2007; Kim et al., 2008; Wollenberg et al., 2008; Xing et al., 2016). SA can initiate flowering through autonomous pathway *via FVE* and *FCA* gene products also, which further regulates the floral integrator gene *FT. FT* mRNA produced in leaves moves through the vasculature towards the SAM and initiates the developmental procedures leading to floral induction (Nelson et al., 2000; Sawa et al., 2007; Song et al., 2014). It has been proved by Shah et al. (2021) that SA ameliorated leaf health- related marker and its profile in *Malus domestica*, and they suggested that plant breeders employ this as a major tool for assessing floral induction character in plants. Martinez et al. (2004) reported that SA promotes flowering by triggering photoperiodic and autonomous pathways. They also noted that SA is a crucial regulator of floral induction in non-stressed plants by acting as an activator of *FT* at the same time as a negative regulator of *FLC* (the floral repressor). Their experiments proved that transgenic *Arabidopsis*, with impaired SA accumulation, showed delayed flowering with respect to its wild-type. Enhanced biosynthesis of SA in the present study due to the endophytic association might have fine tuned the floral induction pathway.

Jasmonic acid (JA) biosynthesis and signalling are a pre-requisite for floral evocation. The JA levels has been reported to be elevated in *Arabidopsis* and rice after co-cultivation with *P. indica* (Stein et al., 2008; Vahabi et al., 2013). Niwa et al. (2018) demonstrated that JA synthesised in growing flower buds induced functional development of floral whorls and triggered petal elongation in tomato. They suggested that JA integrates the signals of other phytohormones such as gibberellins and auxin to determine the timing of flower opening. In the present study, there is a significant increase in cis-OPDA, JA-Ile and JA levels. The elevated levels of phytohormones might have complimented the flowering response and development of floral organs in coordination with other growth phytohormones.

### Effect of *P. indica* on the photoperiod pathway

**Figure 8:**
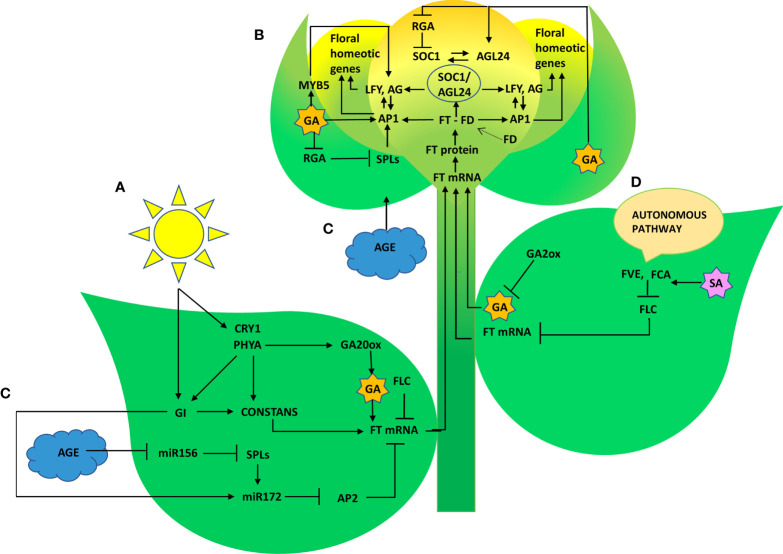
Graphical summary of key regulatory pathways triggered by *P. indica* that induced floral transition in *P. nigrum* L. **(A)** Photoperiod pathway **(B)** Gibberellin biosynthesis pathway **(C)** Age pathway **(D)** Autonomous pathway.

Representative genes of various pathways like photoperiod, autonomous, GA, vernalization, age, and stress response pathways were quantified to figure out the suitable pathway by which *P. indica* prompt early floral induction in *P. nigrum* on various days after root infection. First of all, we tried to find an explanation for the early flowering response of the plant. Whether the response is developmentally regulated by flowering response genes. Floral induction has been defined by Evans (1971) as the sequence of physiological events in response to various stimulus resulting in production of a signalling molecule which is transported to the SAM. The photoperiod induced pathway involves interaction of the two photoreceptor family genes *PHYA* and *CRY1.* These photoreceptors absorbs the periodic red and blue light signals respectively and transmits them to the circadian clock. This in turn synchronizes the expression of the pivotal gene *CO* in the leaf phloem cells by preventing its degradation and conferring protein stability (An et al., 2004; Valverde et al., 2004; Zeevaart, 2008). In the current study, the transcript level of *PHYA* in *P. indica* inoculated plants was 2.12 - fold higher than the control plants and a simultaneous upregulation in the expression of *CO* (8.63-fold) was observed on 50^th^ DAI. *CRY1* is the blue light-absorbing cryptochromes that promote flowering, and its mRNA level was 2.35 -fold higher than the uninoculated control plants (at 60^th^ DAI). The elevation in the expression levels of *PHYA* and *CRY1* resulted in simultaneous upregulation in the expression of *CO*. CO protein level activates the downstream target gene *FT*, which is the signalling molecule that is conveyed to the SAM, to induce flowering (**Figure 8**).

The irreversible biochemical and cellular changes in the SAM in response to floral induction which irreversibly commit the SAM to change from vegetative to reproductive phase is known as floral evocation (Evans, 1971). Upon reception of the mobile signal FT, the SAM becomes committed to flowering. Being the conserved promoter of flowering, FT plays its role in the vasculature of leaves and cotyledons and acts downstream of the flowering regulatory pathways where it works alongside FD (bZIP transcription factor). The FT-FD complex stimulates floral meristem identity genes (*AP1*, *AG*, and *LFY*), which in turn switches on the floral homeotic genes on the flanks of the inflorescence meristem (Guo et al., 1998; Corbesier et al., 2007) resulting in floral evocation (**Figure 8**). The upregulation of *FT* (4.44- fold) and *FD* (3.38- fold) with a simultaneous depression in the level of *FLC* was noted between *P. indica* treated and control plants was observed in the study on the 50^th^ DAI. Repression of *FLC* expression (via *FLC* chromatin modification) enables plants to initiate flowering. *FLC* which is constitutivey expressed in the vegetative tissues are reported to bind to the promoters of *FT, SOC1* and *FD* preventing the expression of these genes, thereby repressing the control of floral induction by the circardian clock (Deng et al., 2011). Downregulation of *FLC* and upregulation of *FT* resulted in a noticeable change in the expression of the floral homeotic genes; *AP1* (8.17- fold), *AG* (5.89-fold) and *LFY* (9.44 - fold) on the 60^th^ DAI. *LFY* gene reduces the floral evocation time and controls the floral transition and floral organ development in coordination with several other genes. AP1 and AG interacts with LFY. LFY (floral meristem identity gene) promotes floral fate by coordinating with the floral commitment factor AP1. High expression of *AP1* (both meristem identity and floral organ identity gene) irreversibly switch the plant to flower fate than enabling branch formation (Jin et al., 2021) responsible for the formation of sepals and petals. *AG* is a floral organ identity gene which codes for the development of stamens and carpels.

GI, a protein that plays a role in the regulation of circadian rhythm and photoperiodic flowering was quantified. It works in a *CO*-independent pathway, triggering photoperiodic flowering by inducing the *FT* gene, and its expression level was appreciably high (9.85- fold) on the 60^th^ DAI. mRNA level of the *FLC* gene was seen downregulated simultaneously. These results shows that the photoperiod pathway genes are positively regulated by *P. indica.*

### Effect of *P. indica* on the Giberellic acid induced pathway

Gibberellins (GA), as a significant hormonal signal in the floral induction pathway, are required for early flowering. The pathway involves bHLH transcription factor *MYB5* as an intermediary, which is responsible for promoting *LFY*. An up-regulation in *MYB5* has been observed on 50^th^ DAI. The expression of the orthologous GA biosynthesis gene *GA20ox2* (positive regulator) and *GA2ox* (negative regulator) were quantified. The expression level was comparable between the experimental and control plants initially. However, *GA20ox2*, the positive regulator was seen upregulated (7.28-fold) in the colonized plants on 60^th^ DAI and *GA2ox* (negative regulator) was downregulated. Furthermore, GA also interacts with *SOC1* by a separate pathway activating the *LFY* and *AP1* genes to induce flowering. A similar report is available which proved colonization by *P. indica* leading to an early flowering response in *Arabidopsis* by activating gibberellin biosynthesis pathway by Kim et al. (2017). Moreover, GA induced signal transmission is DELLA protein degradation-dependent (Cheng et al., 2004). *RGA1* belonging to the DELLA gene family was found to be downregulated simultaneously in the present study. Besides that, *SOC1* and *AGL24* create an autoregulatory feedback loop by binding to each other’s promoters (Liu et al., 2008). The heterodimer (SOC1/AGL24) is necessary for nuclear localization and expression of *LFY* (Lee et al., 2008; Peer et al., 2021). *P. indica* showed its stimulatory effect on the GA responsive flowering integrator genes *AGL24* (6.93 to 9.74- fold in 50 to 60 DAI), and SOC1 (2.98 to 7.68 fold in 40 to 50 DAI) strengthening the idea that the phytohormone GA can also be the target of the fungus in inducing early flowering response.

### Effect of *P. indica* on the autonomous pathway

The autonomous pathway controls over the *FLC* expression level right through the developmental stages independent of photoperiod and GA pathways (Komeda, 2004). An antisense-mediated chromatin silencing mechanism constituted by a set of genes, suppresses the expression of *FLC*, the inhibitor of *SOC1*, to promote flowering (Michaels and Amasino, 2000; Wu et al., 2020). In this study, the key Flowering time control genes, *FVE* which regulates flowering time by repressing *FLC* through histone modification: H_3_K_4_ trimethylation and H_3_ acetylation and *FCA*, which is responsible for the post transcriptional regulation of genes involved in floral induction were also quantified. The genes *FVE* and *FCA* showed significant response to *P. indica* accompanied by a decrease in the transcript level of *FLC* in our study. Regulatory mechanism of these genes and its biological relevance still remains unclear. Anyway, the pathway promotes flowering by alleviating the repressor, *FLC.*

### Effect of *P. indica* on the age pathway

The Age pathway and the photoperiodic pathway act in tandem. The juvenile phase of a plant ends with an increase in the transcript level of *SPL3,-9* in the SAM (Cardon et al., 1999; Rhoades et al., 2002). A temporally regulated miRNA (miR156), copious in seedlings, and its level declines with time, which is mediated by the accumulation of photosynthates (sugars) and also due to degradation of DELLA proteins (Wu and Poethig, 2006; Wu et al., 2009; Yang et al., 2011; Proveniers, 2013; Yu et al., 2013). This developmental decline, enhances the level of its target SPLs, *SPL3*, and *SPL9* in leaves and shoot apical meristem. This induces flowering through activating MADS -box genes, *AP1*, *LFY*, and *SOC1* (Wang et al., 2009a; Yamaguchi et al., 2009). The transcript level of *SPL9* was quantified and was found to have a significant fold change (6.85 to 39.42 -fold) in its expression from 40^th^ to 60^th^ DAI with a simultaneous down regulation in the expression of *AP2* transcript. *AP2* do play a double role, by functioning as the transcriptional activator of *AGAMOUS-LIKE15* (a floral repressor) and at the same time as a repressor of SOC1 (the floral inducer) (Yant et al., 2010). In the present study *AP2* expression is downregulated, so that SOC1 is now activated to induce floral organ identity genes. This is in contrary to the results of Pan et al. (2017). In their experiment to elucidate the targets of the fungus in promoting floral initiation in *Arabidopsis*, they found that the genes involved in age and autonomous pathway are not triggered. Their results suggested that the targets of the endophyte were GA and photoperiod pathways.

The floral transition involves extensive crosstalks, feedback, or feedforward loops between the biomolecules within flowering time regulatory pathways. All four pathways intersect at certain points thereupon eliciting the key floral regulators, *SOC1*, *LFY*, and *AP1* in the meristem and *FT* in the phloem. Expression of these genes, in turn, activates the downstream floral meristem identity genes which are responsible for floral organ development (Wilson et al., 1992). The mRNA level of *AG* was analysed and was found upregulated (5.89 -fold) at 60 DAI.

Vernalization represses the floral repressor gene by epigenetic silencing of *FLC via* histone methylation (Bastow et al., 2004; Searle et al., 2006). In the current study, the planting material was not subjected to vernalization. Hence the vernalization pathway was not analysed. Plants can immediately switch to the reproductive stage as an emergency response by allocating their resources to yield at least some viable seeds when they encounter a stressful situation. This serves as an adaptive mechanism by which plants preserve their species.

### Effect of *P. indica* on the biotic stress induced pathway

In this study, we checked the expression levels of two stress-responsive genes, *WRKY22* and *SID2*. The expression level of these genes was found to be at comparable levels in *P. indica* inoculated and uninoculated control (graph not included). The result suggests that the preponement in flowering was not the result of stress response.

## Conclusion

It has been found that *P. indica* promotes growth and prepones floral induction and evocation in black pepper. The enhancement of phytohormone levels in the inoculated plants is responsible for the growth advancement and plays a crucial role in regulating floral induction. Taken together, the data demonstrated that *P. indica* triggers preponement in floral induction and evocation in black pepper. *P. indica* induced the response in *P. nigrum* by triggering the photoperiodic, GA biosynthesis, autonomous, and age pathways, thereby reducing the juvenile phase of the major spice crop.

## Data availability statement

The raw data supporting the conclusions of this article will be made available by the authors, without undue reservation.

## Author contributions

RSL and EVS conceived the research plans and designed the experiments. RSL performed the experiments and wrote themanuscript. SS participated in data analysis; KNA helped in designing the experiment and made critical revisions in the manuscript. EVS supervised and completed the final edits to the manuscript. All authors contributed to the article and approved the submitted version.

## Funding

National Post-Doctoral Fellowship, Science and Engineering Research Board, Department of Science and Technology, India and the host institute Rajiv Gandhi Centre for Biotechnology for all other support.

## Acknowledgments

The authors sincerely acknowledge the services provided by the Metabolomics Facility, NIPGR, New Delhi, India for the LC-MS/MS analysis, IISER, Trivandrum, India for the SEM analysis and the technical support from the host Institute, Rajiv Gandhi Centre for Biotechnology, India for Confocal microscopy and qRT-PCR analysis. Acknowledging Mr. Akash P., Ph.D Scholar, Transdisciplinary Biology Division for his support in editing the figures.

## Conflict of interest

The authors declare that the research was conducted in the absence of any commercial or financial relationships that could be construed as a potential conflict of interest.

## Publisher’s note

All claims expressed in this article are solely those of the authors and do not necessarily represent those of their affiliated organizations, or those of the publisher, the editors and the reviewers. Any product that may be evaluated in this article, or claim that may be made by its manufacturer, is not guaranteed or endorsed by the publisher.

## Supplementary material

Supplementary Figure 1
**(A)** *P. indica* inoculated plants and control plants *P. nigrum* (Panniyur1) three months post germination. **(B)** *P. indica* colonized plants *P. nigrum* (Panniyur1) and control plants six months post germination. **(C)** *P. nigrum* (Panniyur1) treated and control plant 8 months after transplantation. **(D)** *P. nigrum* (Panniyur1) treated and control plant 15 months after transplantation. **(E)** *P. nigrum* (Panniyur1) treated and control plant 22 months after transplantation.

Supplementary Figure 2 SEM analysis (FEI- NOVA NANO SEM, Columbia) of gold sputter coated *Piriformospora indica* spores in native form grown on the cellophane membrane over PDA medium.
