# Supplementary figures and images for: Root colonization by the endophytic fungus Piriformospora indica shortens the juvenile phase of Piper nigrum L. by fine tuning the floral promotion pathways
Source: Front Plant Sci. 2022 Nov 9;13:954693. doi: 10.3389/fpls.2022.954693 (PMC9720737; doi:10.3389/fpls.2022.954693)

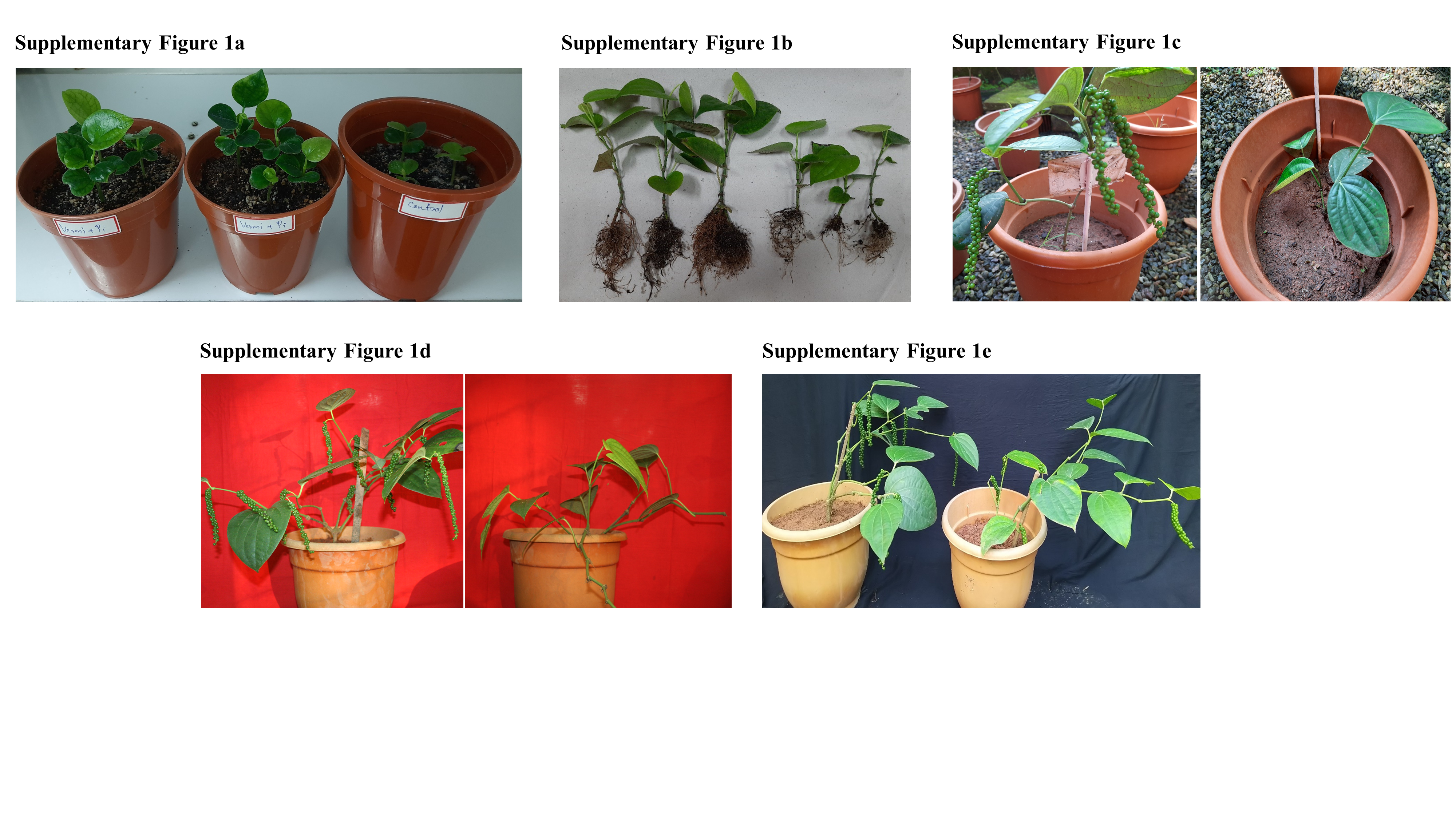

Supplement: Supplementary Figure 1 — (A) P. indica inoculated plants and control plants P. nigrum (Panniyur1) three months post germination. (B) P. indica colonized plants P. nigrum (Panniyur1) and control plants six months post germination. (C) P. nigrum (Panniyur1) treated and control plant 8 months after transplantation. (D) P. nigrum (Panniyur1) treated and control plant 15 months after transplantation. (E) P. nigrum (Panniyur1) treated and control plant 22 months after transplantation. [file Image_1.tif]

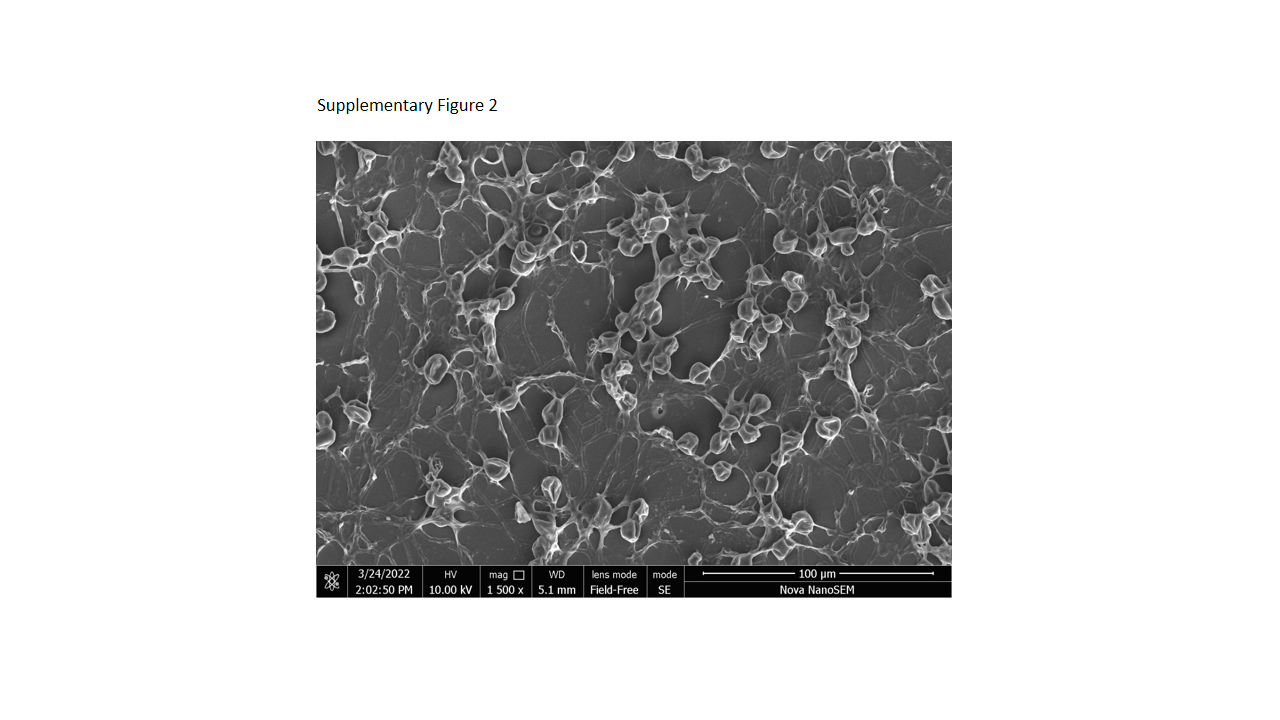

Supplement: Supplementary Figure 2 — SEM analysis (FEI- NOVA NANO SEM, Columbia) of gold sputter coated Piriformospora indica spores in native form grown on the cellophane membrane over PDA medium. [file Image_2.tif]
