# Supplementary material for: Root colonization by the endophytic fungus Piriformospora indica shortens the juvenile phase of Piper nigrum L. by fine tuning the floral promotion pathways
Source: Front Plant Sci. 2022 Nov 9;13:954693. doi: 10.3389/fpls.2022.954693 (PMC9720737; doi:10.3389/fpls.2022.954693)
Supplement: Supplementary file 3 [file Table_1.docx]

| Sl no: | Primer name | Primer sequence (5’-3’) |
| --- | --- | --- |
| 1 | *Pi-TEF* | FP - TCGTCGCTGTCAACAAGATG  RP - ACCGTCTTGGGGTTGTATCC |
| 2 | *PHYA* | FP - TTCCTCCTTGGACTTGGAGA  RP - CAGCGATCAATCCCTCTGAT |
| 3 | *CRY1* | FP - TGTGATAGCCTTTCCCCAAG  RP - GGTCTTCGTGCCTTCTGTTC |
| 4 | *CO* | FP - ATCCCTTCCCTTCCTCACAT  RP - GCTTTGATTGGGGGAAAAAT |
| 5 | *FT* | FP - AGAAGGAAGAAATCGCAGCA  RP - CGGAGGGAGAGAAGAGATCA |
| 6 | *FLC* | FP - CTGCCCTCGTCTTCTCCTC  RP - TGTGTTCATGATAACGCTGGA |
| 7 | *LFY* | FP - AAGAACGGCCTCGACTACCT  RP - TGGGGCATTTCTCTCCTTTA |
| 8 | *AP1* | FP - GGCATATGACAAGGCAGCTA  RP - CAACATCTAAAGCCACTTCTCC |
| 9 | *AG* | FP - TTGAAGAAGACCAGGGAACTG  RP - TCCCTTTGGTAGCAAGATCG |
| 10 | *GI* | FP - CATTGCGACCTCTTACACCA  RP - ACCCATAACACCACCACACC |
| 11 | *MYB5* | FP - TGGCGCACAATATTGAAAGA  RP - GTGACGCTCATGTTTCTCCA |
| 12 | *GA20OX2* | FP - CACTGGCATTCTTCCTTTGC  RP - GTGAAGTCCGGGTACCTCCT |
| 13 | *GA2OX* | FP - ATGGTCTGAGGCCTTCCATA  RP - CGTTCAAGCATCTCACCTCA |
| 14 | *RGA1* | FP - GCCCAGAAGATGGAGCAG  RP - GAGGGGTTGTAGTGGACAG |
| 15 | *AGL24* | FP - ATTGTGCAACCCGTTTCTTC  RP - TTTCGGATGATACCCAAAGC |
| 16 | *FVE* | FP - GATATGGCGAATGAGCGACT  RP - GCTTTCGGAGAGCAAGTCAG |
| 17 | *FCA* | FP - CAGGGACAACCTCTTCCTCA  RP - TGTTGTGGTTGCCTAGGATG |
| 18 | *SPL9* | FP - GTAGGTGGCCTTCAGCAGAG  RP - GAAGACGCTTTCTGCAGCTT |
| 19 | *AP2* | FP - AAATGGTTATCACCCCCTCA  RP - CTCTTTGGGAGTTTGGTTGG |
| 20 | *WRKY22* | FP - CCCAGATGTGAGGTCTTCATAC  RP - GCAGGGTCAGGTAATTGGATAG |
| 21 | *SID2* | FP - TCCTGAACATTTCCAAGATGG  RP - ATGGAGGTCGTCCAGCATAG |
| 22 | *IAA* | FP - TGTCCGGACTCCTACCAGAG  RP - GATGAACCCAGAGGCAAAGA |
| 23 | *IPT* | FP - AATGCAGCTTGTCGGTAAGG  RP - CATGCACGGAGCTACAAAAA |
| 24 | *actin* | FP - AATACCACCCATAAACCAGATAGG  RP - GATGTGGATATCGAAGGCTGAA |

**Table 1: Sequence information of primers used for qRT-PCR analysis**
